# Expression and significance of Her2 and Ki-67 in gastric adenocarcinoma without distant metastasis: a cohort study

**DOI:** 10.1186/s12876-020-01484-9

**Published:** 2020-10-15

**Authors:** Zhijian Wei, Lei Huang, Xinyue Zhang, Aman Xu

**Affiliations:** 1 Department of General Surgery, The First Affiliated Hospital of Anhui Medical University, 218 JiXi Avenue, Hefei, 230022 Anhui China; 2 Department of Academic Research, The First People’s Hospital of Hefei, Hefei, China

**Keywords:** Gastric adenocarcinoma, Human epidermal growth factor receptor 2 (Her2), Nucleus-associated antigen ki-67, Cohort study

## Abstract

**Background:**

The significance of human epidermal growth factor receptor 2 (Her2) and nucleus-associated antigen Ki-67 expression remains controversial in gastric adenocarcinoma (GaC). The aim of this study was to investigate the expression and clinicopathologic and prognostic significance of Her2 and Ki-67 in resected GaC without distant metastasis.

**Methods:**

Malignant tissues and clinicopathologic data were obtained from 195 patients with resected non-metastatic GaC. Immunohistochemistry staining was performed to examine the expression of Her2 and Ki-67; their association with clinicopathologic factors were investigated using logistic regression, and their association with survival was explored using Kaplan–Meier analysis and Cox proportional hazards regression.

**Results:**

Her2 was majorly expressed in cell membrane and Ki-67 in cell nucleus in non-metastatic GaC. Stronger Her2 expression was significantly associated with better tumor differentiation, neurovascular invasion, less advanced pathological tumor (pT) stage, and more advanced pathological node (pN) stage; while Ki-67 expression was not significantly associated with any investigated clinicopathologic factors. Patients with both negative Her2 and negative Ki-67 expression had poorer tumor differentiation, and more advanced pT and pathological tumor-node-metastasis (pTNM) stages; the association with pT and pTNM stages were further confirmed by multivariable analyses, especially in node-negative disease. Her2 or Ki-67 alone was not significantly associated with pTNM stage. A strongly positive (+++) Her2 expression was associated with poorer survival in multivariable analysis only (*P* = 0.047); while Ki-67 or combined expression was not significantly associated with prognosis.

**Conclusions:**

In non-metastatic GaC, Her2 expression and combined expression of Her2 and Ki-67 were associated with several clinicopathologic factors including tumor differentiation and stage, and only a +++ Her2 expression was associated with poorer prognosis in multivariable analysis with marginal significance in this study; while Ki-67 alone had both limited clinicopathologic and prognostic values.

## Background

Gastric cancer, the majority of which is gastric adenocarcinoma (GaC), is one of the most prevalent and lethal digestive malignancies worldwide [1, 2]. Although the diagnosis and treatment modalities have improved, the overall 5-year survival rates of GaC remain poor [3–5]. GaC is highly malignant, invasive, and chemo-resistant, and rapidly progressive. Recurrence and metastasis occur frequently after surgery and chemo(radio)therapy, which impacts patients’ survival time and quality of life greatly. Tumor-node-metastasis (TNM) stage well differentiates prognoses and guides therapy in GaC [3–5].

The occurrence and development of GaC is a multi-stage process which involves multiple gene dysregulations (oncogene activation and tumor-suppressor gene inactivation) and the resulting changes in cell biology behaviors [6, 7]. Human epidermal growth factor receptor 2 (Her2), which belongs to the ErbB/Her family of receptor tyrosine kinases, is an oncogene and predictive biomarker in various cancers [8, 9]. Nucleus-associated antigen Ki-67 is an important cell proliferation-associated protein marker, and its expression is correlated with invasion depth, lymphatic metastasis, and differentiation grade in various solid tumors, especially in colorectal cancer, breast cancer, and liver carcinoma [10–12].

Her2 is commonly tested for metastatic GaC. These two markers have also been included in pathology report for resected GaC in China. The association of Her2 and Ki-67 expression with clinicopathologic factors including tumor pathological tumor-node-metastasis (pTNM) and individual pathological tumor (pT) or pathological node (pN) stages and differentiation and their prognostic roles in non-metastatic GaC remains controversial [13, 14]. This study aimed at investigating the expression of Her2 and Ki-67 in resected GaC without distant metastasis, and at revealing their clinicopathologic and prognostic values.

## Methods

### Patients and specimens

A total of 210 GaC patients undergoing surgical resection between October 2013 and December 2014 in the First Affiliated Hospital of Anhui Medical University where yearly the greatest number of GaC patients receive resection [15] were potential candidates for this study. The inclusion criteria were: (1) the patient had not received any pre-surgical chemo-, radio-, or bio-therapy; (2) diagnosis of GaC was confirmed by postsurgical pathological examination of resected specimens by at least two experienced pathologists, which was consistent with preoperational pathology; (3) the patient underwent open radical total or partial D2 gastrectomy as appropriate; and (4) all cancers were adenocarcinomas. The exclusion criteria included: (1) the patient had serious comorbidities; (2) the patient had other malignancies simultaneously; (3) the pathological diagnosis was controversial; (4) the patient had previous gastric malignancies; and (5) the patient had received chemotherapy or radiotherapy before resection. In our institution in China, it is not the clinical routine that patients with gastric or cardia cancers receive preoperative treatment. Finally 195 GaC patients were eligible for investigation (Table 1). The resected GaC specimens were collected after resection. Basic patient characteristics were recorded. The clinicopatholgic data were obtained from the surgery and pathology records. Disease stage was classified or recoded according to the American Joint Committee on Cancer/Union for International Cancer Control TNM staging system, seventh edition [16]. Written informed consent was obtained from each participating individual, and our study was approved by the local Institutional Review Board and carried out following the Declaration of Helsinki [17] and Good Clinical Practice guidelines [18].

**Table 1:** Clinicopathologic features of enrolled patients with non-metastatic gastric cancer (n = 195)

Variable		Value
Age (years)	As continuous	64 ± 9; 64 (58–71)
Age group	< 60 years	58 (30)
	60–69 years	82 (42)
	≥ 70 years	55 (28)
Sex	Male	148 (76)
	Female	47 (24)
Tumor location	Gastric cardia and fundus	102 (52)
	Gastric body	38 (20)
	Gastric antrum and pylorus	55 (28)
Differentiation grade	Well/moderate	48 (25)
	Poor/undifferentiated	147 (75)
Neurovascular invasion	Yes	35 (18)
	No	160 (82)
Cancer embolus	Yes	39 (20)
	No	156 (80)
Tumor length (cm)	As continuous	4.8 ± 2.2; 4.5 (3.0–6.0)
Tumor width (cm)	As continuous	3.7 ± 1.8; 3.5 (2.5–4.5)
Harvested lymph node	As continuous	19 ± 7; 18 (15–22)
Metastatic lymph node	As continuous	4 ± 5; 2 (0–6)
Lymph node ratio	As continuous	0.20 ± 0.24; 0.10 (0.00–0.32)
pT stage	1	20 (10)
	2	23 (12)
	3–4	152 (78)
pN stage	0	70 (36)
	1	34 (17)
	2	43 (22)
	3	48 (25)
pTNM stage	IA	17 (9)
	IB	14 (7)
	IIA	5 (3)
	IIB	44 (23)
	IIIA	32 (16)
	IIIB	35 (18)
	IIIC	48 (25)
Ki-67 and Her2 expression	Her2 negative and Ki-67 negative	49 (25)
	Her2 negative and Ki-67 positive	91 (47)
	Her2 positive and Ki-67 negative	21 (11)
	Her2 positive and Ki-67 positive	34 (17)

Categorical data are shown as count (percentage [%]), and continuous data as mean ± standard deviation; median (interquartile range)

*pT stage* pathological tumor stage, *pN stage* pathological node stage, *pTNM stage* pathological tumor-node-metastasis stage, *Her2* human epidermal growth factor receptor 2

### Reagents

Anti-Her2 monoclonal antibody was purchased from the Fuzhou New Biotechnology Development company. The other primary antibodies including the mouse-anti-human Ki-67 monoclonal antibody working solution (MAB-0129), the ready-to-use non-biotin immunohistochemistry streptavidin-peroxidase detection kit (KIT-9902), the diaminobenzidine chromogenic reagent (DAB-2301), the citrate tissue-antigen recovery solution (MVS-0100), and the neutral gum were all obtained from the Maixin Biotechnology Co. Ltd., Fuzhou. The reagents were all stored at 4 °C before use.

### Immunohistochemistry

The obtained tissues were first hematoxylin–eosin-stained to be confirmed as GaC tissues. The protein marker expression in GaC tissues was detected using immunohistochemistry (streptavidin-peroxidase). As standard, automated platform for immunohistochemistry was used. The staining method has been previously described [19], and the major procedures were: (1) the slides were immersed in 10% silver nitrate solution for 24 h, and then dehydrated in 95% ethanol, followed by water rinsing and air drying. Then they were processed by 2% APES acetone for 30 s, followed by wash with pure acetone and airing; (2) the specimens were fixed in 10% formaldehyde, embedded in paraffin, serially sectioned into 3 μm-thick slices, attached to slides, dewaxed by xylene, and hydrated by gradient ethanol, followed by phosphate-buffered saline (PBS) wash and airing; (3) antigen recovery was conducted using citrate with high temperature and pressure, followed by cooling and water and PBS wash. Then the sections were incubated in 3% hydrogen peroxide deionized water for 5–10 min to inactivate the endogenous peroxidase, followed by water and PBS wash; (4) the Her2 or Ki-67 (working solution) monoclonal antibodies (50 μL) was added to the specimens respectively, followed by incubation at 4° C overnight or at room temperature for 1 h and then PBS wash; (5) the polymer enhancer (50 μL) was added followed by incubation at room temperature for 20 min and then PBS wash. Then the general-type Immunoglobulin G antibody-Horseradish Peroxidase polymer (anti-mouse/rabbit, 50 μL) was added followed by incubation at room temperature for 15–30 min and then PBS wash; (6) the sections underwent color-developing in diaminobenzidine solution (100 μL/section) for 2–8 min with microscopic observation, followed by distilled water rinse for 10 min to stop the reaction and then hematoxylin counterstain for 3 min plus water wash; and (7) the sections were acidized using 1% hydrochloric acid and processed by saturated lithium carbonate solution, followed by rinse. Then the sections were gradient alcohol-hydrated, xylene-hyalinized, and neutral gum-sealed, followed by microscopic observation. The positive tissue section in the pre-experiment set was selected as the positive control, and the negative control was obtained by replacing the primary antibody with PBS.

### Assessment criteria

The stained sections were evaluated by 2 experienced pathologists in a blind way. Observed in high power field (× 400), randomly 5 fields were selected and examined for each section, and the number and intensity of positively-stained cells were recorded and averaged, with 100 cells in each field counted respectively. In case of a discrepancy, a third senior investigator was consulted and agreement was reached by consensus. The positivity level of Her2 expression follows the National Comprehensive Cancer Network (NCCN) Guidelines (https://www.nccn.org): − means “no reactivity or membranous reactivity in < 10% of cancer cells”; + means “faint or barely perceptible membranous reactivity in ≥ 10% of cancer cells; cells are reactive only in part of their membrane”; ++ means “weak to moderate complete, basolateral or lateral membranous reactivity in ≥ 10% of cancer cells”; +++ means “strong complete, basolateral, or lateral membranous reactivity in ≥ 10% of cancer cells”. – or + expression indicates a negative result, and +++ expression suggests a positive finding. In case of ++ expression by immunohistochemistry, in situ hybridization was further performed, and cases with an average Her2 copy number ≥ 6.0 signals/cell were considered positive. For Ki-67, the results were assessed according to the percentage of positive cells: − means very low proliferation activity with proportion of Ki-67-positive cells < 25%; + means low proliferation activity with proportion of Ki-67-positive cells 25–50%; ++ means moderate proliferation activity with proportion of Ki-67-positive cells 50–75%; +++ means high proliferation activity with proportion of Ki-67-positive cells > 75%. A section was regarded negative in Ki-67 expression with < 50% positive cells, and positive with ≥ 50% positive cells following relevant reports published recently [20–24]. Cancers were further categorized into 4 groups according to combined expression of both markers: double negative, Ki-67 positive and Her2 negative, Her2 positive and Ki-67 negative, and double positive.

### Statistical analysis

Continuous variables were presented as mean ± standard deviation; median (interquartile range), and categorical variables as count (percentage [%]). Clinicopathologic factors in different subgroups by the combined expression of Her2 and Ki-67 were compared using the *χ*^2^ or analysis of variance test according to data type. Logistic regression was used to determine the association of marker expression with other clinicopathologic factors. Kaplan–Meier survival curves stratified by marker expression were plotted, and prognostic differences across groups were examined using the log-rank test. The association of marker expression with survival was further examined using univariable and multivariable Cox proportional hazards regression. Data were analyzed using the R (v. 3.4.0, Vienna, Austria) statistical software (https://www.r-project.org/). A finding was statistically significant with a 2-sided *P* value < 0.05.

## Results

### Patient characteristics

For the 195 analyzed patients (Table 1), the mean age was 64 years, and males took up 76%. Most of the tumors were located at gastric cardia and/or fundus (52%), and were poorly-differentiated or undifferentiated (75%). The mean tumor length was 4.8 cm. The mean metastatic lymph node number was 4 based on an average harvest of 19 lymph nodes per patient, and the mean positive-harvested lymph node ratio was 0.20.

### Expression of Her2 and Ki-67 and their association with clinicopathologic factors in GaC

**Fig. 1:**
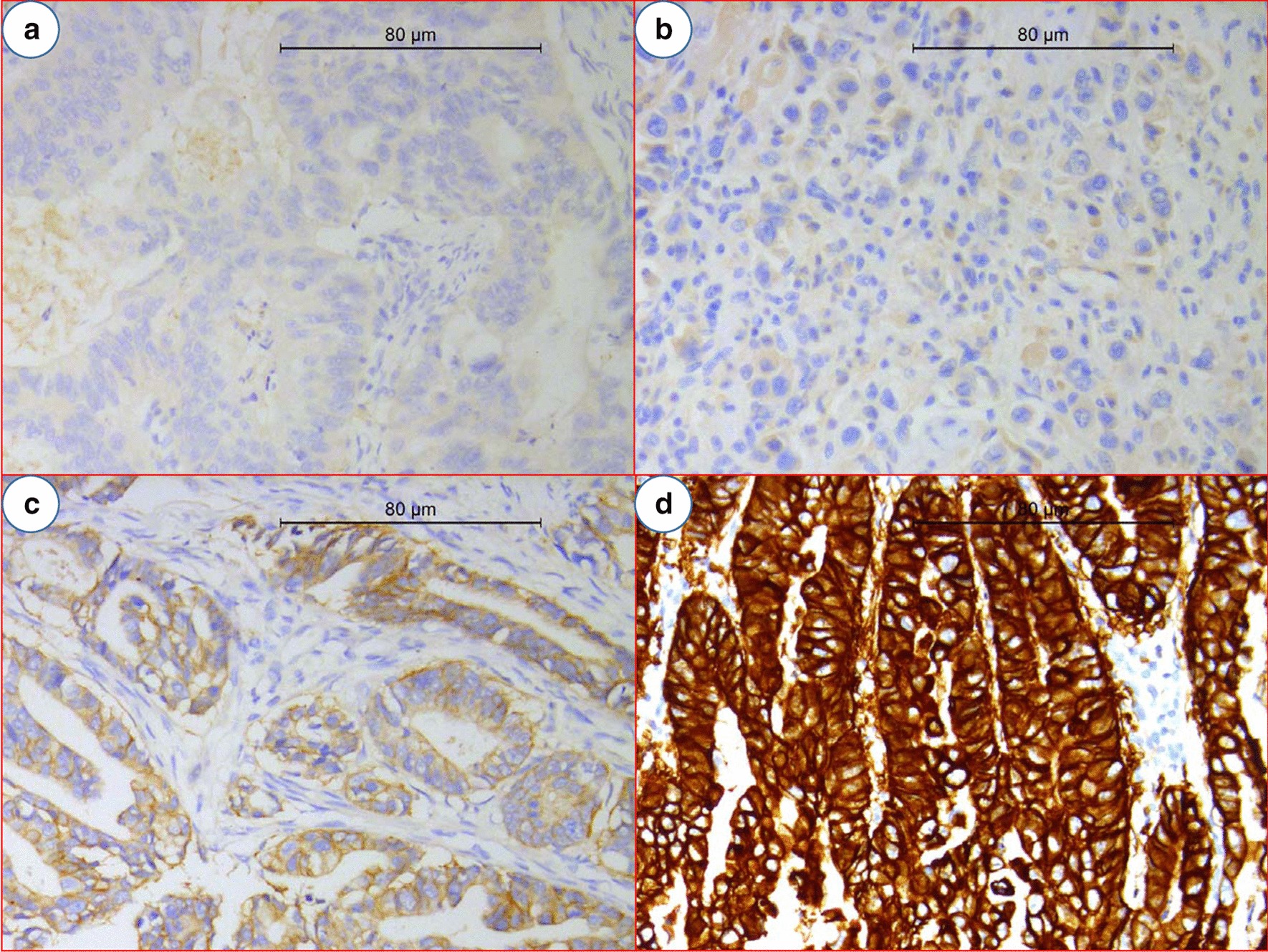
Expression of Her2. **a** pTNM stage I gastric adenocarcinoma; **b** + from pTNM stage III gastric adenocarcinoma; **c** ++ from pTNM stage II gastric adenocarcinoma; **d** +++ from pTNM stage II gastric adenocarcinoma. The positivity degree of Her2 expression follows the National Comprehensive Cancer Network guidelines (https://www.nccn.org): − means “no reactivity or membranous reactivity in < 10% of cancer cells”; + means “faint or barely perceptible membranous reactivity in ≥ 10% of cancer cells; cells are reactive only in part of their membrane”; ++ means “weak to moderate complete, basolateral or lateral membranous reactivity in ≥ 10% of cancer cells”; +++ means “strong complete, basolateral, or lateral membranous reactivity in ≥ 10% of cancer cells”. *Her2*, human epidermal growth factor receptor 2

**Fig. 2:**
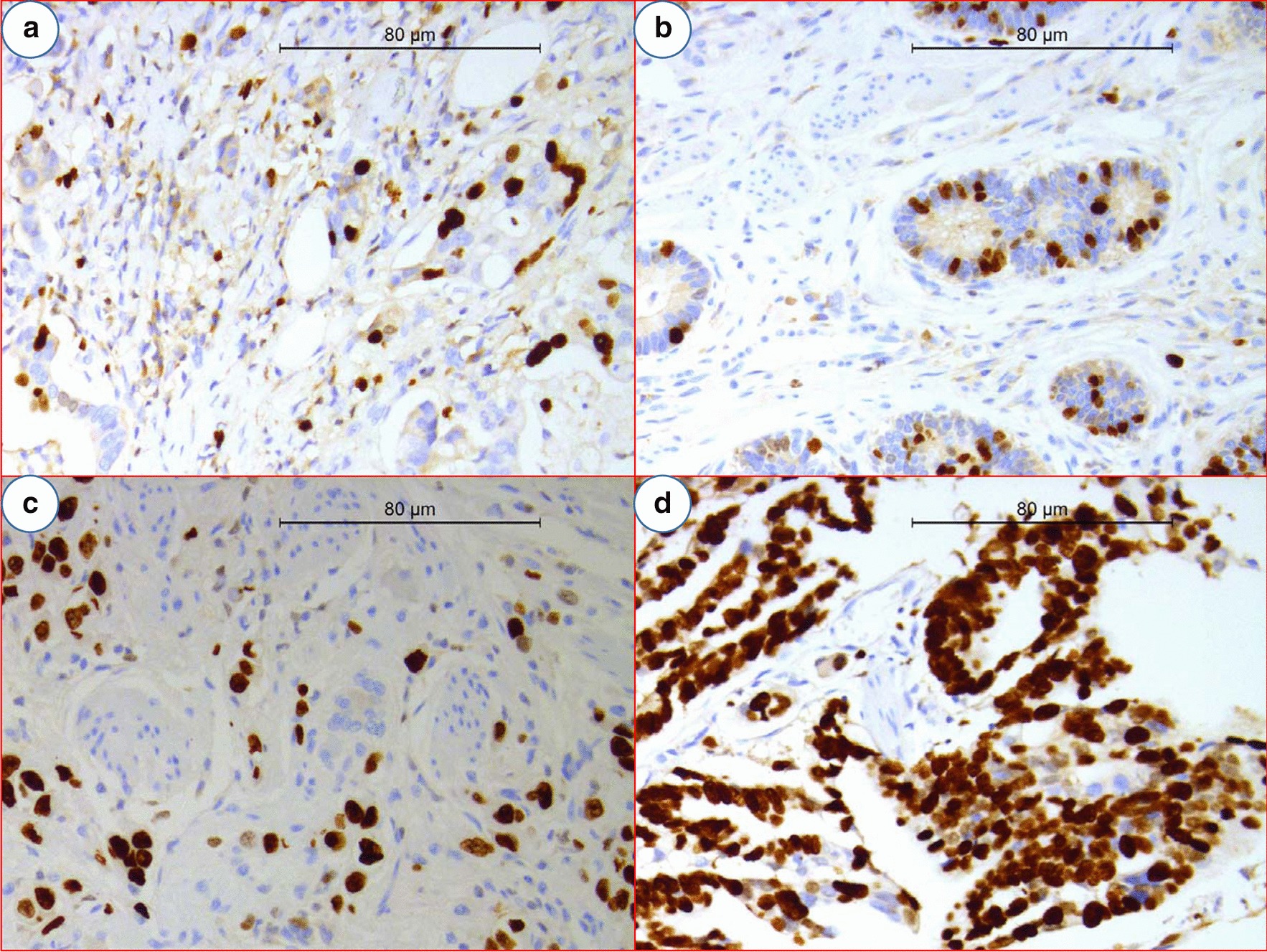
Expression of Ki-67. **a** from pTNM stage I gastric adenocarcinoma; **b** from pTNM stage II gastric adenocarcinoma; **c** ++ from pTNM stage I gastric adenocarcinoma; **d** +++ from pTNM stage III gastric adenocarcinoma. For Ki-67, − means very low proliferation activity with proportion of Ki-67-positive cells < 25%; + means low proliferation activity with proportion of Ki-67-positive cells 25–50%; ++ means moderate proliferation activity with proportion of Ki-67-positive cells 50–75%; +++ means high proliferation activity with proportion of Ki-67-positive cells > 75%

Both Her2 and Ki-67 were presented as brown or yellow granules in GaC. Her2 was majorly expressed in cell membrane and the positive expression proportion was 28% (Fig. 1). Ki-67 was located at cell nucleus, and the positive proportion was 64% (Fig. 2). Stronger Her2 expression was associated with poorer tumor differentiation, neurovascular invasion, earlier pT stage, and more advanced pN stage; however, expression of Ki-67 was not significantly associated with the investigated clinicopathologic variables (Table 2). Expression of Her2 or Ki-67 was not significantly associated with overall pTNM stage (Table 3).

**Table 2:** Association of clinicopathologic parameters with Her2 and Ki-67 expression using multivariable-adjusted logistic regression

Variable	Her2		Ki-67	
	OR (95% CI)	*P*	OR (95% CI)	*P*
Sex				
Male	1 (reference)		1 (reference)	
Female	0.72 (0.36–1.46)	0.360	0.75 (0.39–1.47)	0.405
Age (as continuous)	1.01 (0.97–1.04)	0.705	1.03 (1.00–1.06)	0.092
Tumor location				
Gastric cardia and fundus	1 (reference)		1 (reference)	
Gastric body	1.59 (0.75–3.37)	0.544	1.51 (0.72–3.17)	0.983
Gastric antrum and pylorus	1.05 (0.51–2.20)	0.647	1.05 (0.52–2.13)	0.367
Tumor differentiation				
Well/moderate	1 (reference)		1 (reference)	
Poor/undifferentiated	*0.25* (0.12–0.53)	< *0.001*	1.10 (0.53–2.26)	0.806
Neural invasion				
No	1 (reference)		1 (reference)	
Yes	2.35 (1.01–5.48)	*0.048*	0.75 (0.33–1.70)	0.487
Tumor thrombosis				
No	1 (reference)		1 (reference)	
Yes	1.24 (0.56–2.75)	0.601	0.96 (0.44–2.08)	0.917
Tumor length (as continuous)	1.09 (0.93–1.29)	0.276	0.97 (0.83–1.13)	0.700
pT stage				
1	1 (reference)		1 (reference)	
2	1.39 (0.41–4.67)	0.134	0.94 (0.27–3.20)	0.559
3–4	*0.26* (0.08–0.85)	*0.001*	0.50 (0.16–1.54)	0.128
pN stage				
0	1 (reference)		1 (reference)	
1	*2.69* (1.09–6.62)	*0.030*	1.42 (0.61–3.30)	0.673
2	*2.71* (1.14–6.43)	*0.027*	1.20 (0.54–2.66)	0.867
3	*2.83* (1.17–6.87)	*0.016*	1.46 (0.65–3.31)	0.559

Her2 and Ki-67 are in the sequential order of −, +, ++ , and +++. The positivity degree of Her2 expression follows the National Comprehensive Cancer Network (NCCN) Guidelines (https://www.nccn.org): − means “no reactivity or membranous reactivity in < 10% of cancer cells”; + means “faint or barely perceptible membranous reactivity in ≥ 10% of cancer cells; cells are reactive only in part of their membrane”; ++ means “weak to moderate complete, basolateral or lateral membranous reactivity in ≥ 10% of cancer cells”; +++ means “strong complete, basolateral, or lateral membranous reactivity in ≥ 10% of cancer cells”. – or + expression indicates a negative result, and +++ expression suggests a positive finding. In case of ++ expression by immunohistochemistry, in situ hybridization was further performed, and cases with an average Her2 copy number ≥ 6.0 signals/cell are considered positive. For Ki-67, − means very low proliferation activity with proportion of Ki-67-positive cells < 25%; + means low proliferation activity with proportion of Ki-67-positive cells 25–50%; ++ means moderate proliferation activity with proportion of Ki-67-positive cells 50–75%; +++ means high proliferation activity with proportion of Ki-67-positive cells > 75%. A section was regarded negative in Ki-67 expression with < 50% positive cells, and positive with ≥ 50% positive cells. Significant associations are shown in italics

*pT stage* pathological tumor stage, *pN stage* pathological node stage, *Her2* human epidermal growth factor receptor 2; *OR* odds ratio, *CI* confidence interval

**Table 3:** Association of Ki-67 and Her2 expression with pTNM stage in patients with non-metastatic gastric cancer

Variable	pTNM stage			*χ*^*2*^/*F*	*P*^a^	Univariable test^b^		Multivariable test^c^	
	I (n = 31)	II (n = 49)	III (n = 115)			OR (95% CI)	*P*	OR (95% CI)	*P*
Ki-67									
Proportion of Ki-67-positive cells (%)	55 ± 21; 60 (50–70)	51 ± 18; 50 (40–60)	53 ± 22; 60 (40–70)	0.44	0.645	1.00 (0.99–1.01)	0.826	1.00 (0.98–1.01)	0.550
−	4 (13)	5 (10)	12 (10)	0.53	0.469	1 (reference)		1 (reference)	
+	3 (10)	14 (29)	32 (28)			1.60 (0.58–4.43)	0.220	1.15 (0.35–3.79)	0.502
++	18 (58)	26 (53)	54 (47)			0.95 (0.38–2.36)	0.375	0.75 (0.26–2.19)	0.381
+++	6 (19)	4 (8)	17 (15)			1.15 (0.38–3.52)	0.996	0.88 (0.25–3.14)	0.869
Negative	7 (23)	19 (39)	44 (38)	1.85	0.174	1 (reference)		1 (reference)	
Positive	24 (77)	30 (61)	71 (62)			0.71 (0.40–1.27)	0.251	0.71 (0.36–1.38)	0.310
Her2									
−	17 (55)	23 (47)	62 (54)	1.41	0.236	1 (reference)		1 (reference)	
+	3 (10)	8 (16)	27 (23)			1.68 (0.76–3.72)	0.338	1.89 (0.74–4.81)	0.169
++	7 (23)	10 (20)	16 (14)			0.64 (0.30–1.35)	0.220	0.57 (0.24–1.35)	0.335
+++	4 (13)	8 (16)	10 (9)			0.62 (0.26–1.48)	0.250	1.68 (0.60–4.72)	0.338
Negative	20 (65)	31 (63)	89 (77)	3.38	0.066	1 (reference)		1 (reference)	
Positive	11 (35)	18 (37)	26 (23)			0.55 (0.30–1.00)	0.051	0.78 (0.38–1.57)	0.482

*pTNM stage* pathological tumor-node-metastasis stage, *Her2* human epidermal growth factor receptor 2, *OR* odds ratio, *CI* confidence interval

^a^Categorical data are shown as count (percentage [%]), and continuous data as mean ± standard deviation; median (interquartile range). The positivity degree of Her2 expression follows the National Comprehensive Cancer Network (NCCN) Guidelines (https://www.nccn.org): − means “no reactivity or membranous reactivity in < 10% of cancer cells”; + means “faint or barely perceptible membranous reactivity in ≥ 10% of cancer cells; cells are reactive only in part of their membrane”; ++ means “weak to moderate complete, basolateral or lateral membranous reactivity in ≥ 10% of cancer cells”; +++ means “strong complete, basolateral, or lateral membranous reactivity in ≥ 10% of cancer cells”. – or + expression indicates a negative result, and +++ expression suggests a positive finding. In case of ++ expression by immunohistochemistry, in situ hybridization was further performed, and cases with an average Her2 copy number ≥ 6.0 signals/cell are considered positive. For Ki-67, − means very low proliferation activity with proportion of Ki-67-positive cells < 25%; + means low proliferation activity with proportion of Ki-67-positive cells 25–50%; ++ means moderate proliferation activity with proportion of Ki-67-positive cells 50–75%; +++ means high proliferation activity with proportion of Ki-67-positive cells > 75%. A section was regarded negative in Ki-67 expression with < 50% positive cells, and positive with ≥ 50% positive cells

^b^Performed using univariable logistic regression

^c^Performed using multivariable logistic regression adjusting for sex, age, tumor location, length, and differentiation grade

### Association of combined expression of both markers with clinicopathologic factors in GaC

Based on the combined expression of both markers, cancers were categorized into four groups (Table 4): double negative (n = 49, 25%), Ki-67 positive and Her2 negative (n = 91, 47%), Her2 positive and Ki-67 negative (n = 21, 11%), and double positive (n = 34, 17%). While no significant differences in patient age, sex, tumor location, size, metastatic lymph node number, lymph node ratio, neurovascular invasion, cancer embolus, or pN stage were observed between the four groups, there existed significant inter-group differences in tumor differentiation (*P* = 0.001), pT stage (*P* = 0.009), and pTNM stage (*P* = 0.022). Patients with negative expression of both markers had highest proportions of poorly-differentiated/undifferentiated tumors (86%), pT stage 3–4 (94%), and pTNM stage III (69%).

**Table 4:** Association of Her2 and Ki-67 expression with clinicopathologic parameters in patients with non-metastatic gastric cancer

Variable		Double negative, n = 49	Ki-67 positive, n = 91	Her2 positive, n = 21	Double positive, n = 34	*χ*^2^/*F*	*P*^a^
Age (years)	As continuous	63 ± 11; 63 (56–70)	63 ± 8; 65 (59–69)	63 ± 10; 63 (55–71)	66 ± 8; 67 (61–73)	0.70	0.554
Age group	< 60 years	19 (39)	25 (28)	7 (33)	7 (21)	1.98	0.160
	60–69 years	17 (35)	45 (50)	6 (29)	14 (41)		
	≥ 70 years	13 (27)	21 (23)	8 (38)	13 (38)		
Sex	Male	37 (76)	66 (73)	15 (71)	30 (88)	0.77	0.379
Tumor location	Gastric cardia and fundus	26 (53)	48 (53)	12 (57)	16 (47)	< 0.01	0.985
	Gastric body	8 (16)	16 (18)	5 (24)	9 (27)		
	Gastric antrum and pylorus	15 (31)	27 (29)	4 (19)	9 (27)		
Differentiation grade	Well/Moderate	7 (14)	18 (20)	9 (43)	14 (41)	11.01	*0.001*
	Poor/undifferentiated	42 (86)	73 (80)	12 (57)	20 (59)		
Neural invasion	Yes	10 (20)	12 (13)	6 (29)	7 (21)	0.44	0.506
Cancer embolus	Yes	10 (20)	20 (22)	4 (19)	5 (15)	0.12	0.733
Tumor length (cm)	As continuous	5.1 ± 2.1; 5.0 (3.5–6.5)	4.8 ± 2.3; 4.5 (3.0–6.0)	4.7 ± 2.6; 4.0 (3.0–5.0)	4.4 ± 2.1; 4.0 (2.8–5.5)	0.59	0.625
Tumor width (cm)	As continuous	4.0 ± 1.6; 4.0 (2.5–5.0)	3.8 ± 1.9; 3.5 (2.5–5.0)	3.6 ± 2.0; 3.0 (2.8–4.5)	3.3 ± 1.5; 3.0 (2.5–4.0)	0.84	0.474
Metastatic lymph node	As continuous	4 ± 5; 3 (0–7)	4 ± 5; 2 (0–7)	3 ± 5; 1 (0–6)	3 ± 4; 2 (0–5)	0.50	0.683
Lymph node ratio	As continuous	0.22 ± 0.23; 0.18 (0.00–0.32)	0.20 ± 0.25; 0.09 (0.00–0.35)	0.18 ± 0.26; 0.05 (0.00–0.28)	0.19 ± 0.24; 0.11 (0.00–0.27)	0.16	0.921
pT stage	1	2 (4)	11 (12)	3 (14)	4 (12)	6.88	*0.009*
	2	1 (2)	10 (11)	4 (19)	8 (24)		
	3–4	46 (94)	70 (77)	14 (67)	22 (65)		
pN stage	0	16 (33)	33 (36)	9 (43)	12 (35)	0.52	0.472
	1	7 (14)	16 (18)	5 (24)	6 (18)		
	2	13 (27)	19 (21)	2 (10)	9 (26)		
	3	13 (27)	23 (25)	5 (24)	7 (21)		
pTNM stage	I	2 (4)	18 (20)	5 (24)	6 (18)	5.24	*0.022*
	II	13 (27)	18 (20)	6 (29)	12 (35)		
	III	34 (69)	55 (60)	10 (48)	16 (47)		

Categorical data are shown as count (percentage [%]), and continuous data as mean ± standard deviation; median (interquartile range)

*pTNM stage* pathological tumor-node-metastasis stage, *Her2* human epidermal growth factor receptor 2

^a^For comparison across groups

The above findings appeared contradictory to the well-known knowledge that strong expressions of Her2 and Ki-67 are both predictors of poor survival, and we further explored the association of the combined expression with pTNM stage in overall, node-negative, and node-positive GaCs and with pT and pN stages using logistic regression. Interestingly, in node-negative disease, negative expression of both markers was significantly associated with the most advanced pTNM stage; however, in overall or node-positive GaC, no significant association of combined expression of the two markers with pTNM stage was observed (Table 5). We further investigated the association of the combined expression of both markers with individual pT and pN stages (Table 6), and performed univariable analysis and 2 sets of multivariable analyses (one with pN and pT stage excluded from covariates, and the other with pN or pT stage included). All models revealed that cancers with negative expression of both markers had the most advanced pT stage; while the combined expression was not significantly associated with pN stage.

**Table 5:** Association of combined Ki-67 and Her2 expression with pTNM stage in patients with non-metastatic gastric cancer estimated using univariable and multivariable-adjusted logistic regression

Ki-67 and Her2 expression	Overall cancer		Node-negative cancer		Node-positive cancer	
	OR (95% CI)	*P*	OR (95% CI)	*P*	OR (95% CI)	*P*
Univariable test^a^						
Her2 negative and Ki-67 negative	1 (reference)		1 (reference)		1 (reference)	
Her2 negative and Ki-67 positive	0.70 (0.38–1.30)	0.500	*0.11* (0.02–0.54)	*0.009*	0.99 (0.45–2.17)	0.109
Her2 positive and Ki-67 negative	0.43 (0.17–1.06)	0.233	*0.13* (0.02–0.84)	*0.033*	0.48 (0.14–1.59)	0.445
Her2 positive and Ki-67 positive	0.48 (0.22–1.05)	0.337	*0.14* (0.02–0.83)	*0.038*	0.39 (0.15–1.05)	0.122
Multivariable test^b^						
Her2 negative and Ki-67 negative	1 (reference)		1 (reference)		1 (reference)	
Her2 negative and Ki-67 positive	0.84 (0.44–1.60)	0.807	*0.07* (0.01–0.56)	*0.025*	1.27 (0.55–2.94)	0.126
Her2 positive and Ki-67 negative	0.64 (0.24–1.70)	0.508	*0.05* (< 0.01–0.69)	*0.030*	0.66 (0.17–2.55)	0.632
Her2 positive and Ki-67 positive	0.76 (0.33–1.75)	0.838	*0.06* (0.01–0.63)	*0.026*	0.55 (0.19–1.63)	0.266

Significant associations are shown in italics

*pTNM stage* pathological tumor-node-metastasis stage, *Her2* human epidermal growth factor receptor 2, *OR* odds ratio, *CI* confidence interval

^a^Performed using univariable logistic regression

^b^Performed using multivariable logistic regression with adjustment for age, sex, tumor location, length, and differentiation grade

**Table 6:** Association of combined Ki-67 and Her2 expression with pT and pN stages in patients with non-metastatic gastric cancer estimated using univariable and multivariable-adjusted logistic regression

Ki-67 and Her2 expression	Association with pT		Association with pN	
	OR (95% CI)	*P*	OR (95% CI)	*P*
Univariable test^a^				
Her2 negative and Ki-67 negative	1 (reference)		1 (reference)	
Her2 negative and Ki-67 positive	*0.22* (0.06–0.76)	*0.035*	0.84 (0.45–1.58)	0.819
Her2 positive and Ki-67 negative	*0.14* (0.03–0.59)	*0.012*	0.62 (0.24–1.57)	0.404
Her2 positive and Ki-67 positive	*0.13* (0.03–0.51)	*0.009*	0.81 (0.37–1.78)	0.985
Multivariable test 1^b^	Model 1		Model 2	
Her2 negative and Ki-67 negative	1 (reference)		1 (reference)	
Her2 negative and Ki-67 positive	*0.14* (0.03–0.61)	*0.044*	1.08 (0.56–2.08)	0.673
Her2 positive and Ki-67 negative	*0.11* (0.02–0.64)	*0.029*	1.21 (0.44–3.34)	0.942
Her2 positive and Ki-67 positive	*0.07* (0.01–0.39)	*0.025*	1.48 (0.62–3.55)	0.429
Multivariable test 2^c^	Model 3		Model 4	
Her2 negative and Ki-67 negative	1 (reference)		1 (reference)	
Her2 negative and Ki-67 positive	*0.13* (0.03–0.64)	*0.039*	1.27 (0.65–2.49)	0.601
Her2 positive and Ki-67 negative	*0.07* (0.01–0.48)	*0.015*	1.64 (0.57–4.74)	0.706
Her2 positive and Ki-67 positive	*0.06* (0.01–0.37)	*0.009*	2.01 (0.80–5.00)	0.263

Significant associations are shown in italics

*pT stage* pathological tumor stage, *pN stage* pathological node stage, *Her2* human epidermal growth factor receptor 2, *OR* odds ratio, *CI* confidence interval

^a^Performed using univariable logistic regression

^b^Performed using multivariable logistic regression with adjustment for age, sex, tumor location, length, and differentiation grade

^c^pN stage was additionally adjusted for in Model 3, and pT stage in Model 4

### The prognostic significance of Her2 and Ki-67

**Fig. 3:**
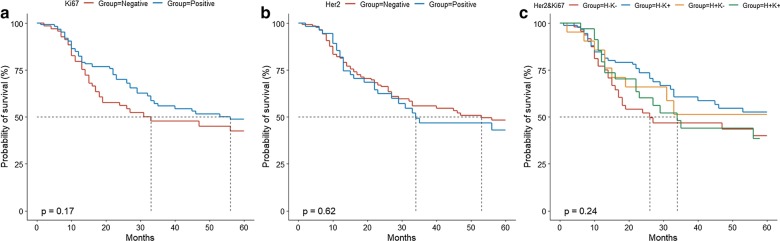
Kaplan–Meier survival curves according to Ki-67 (**a**), Her2 (**b**), and combined expression (**c**). Prognostic differences across groups were examined using the log-rank test. Her2, human epidermal growth factor receptor 2; H–K−, Her2 negative and Ki-67 negative; H–K+, Her2 negative and Ki-67 positive; H+K−, Her2 positive and Ki-67 negative; H+K+, Her2 positive and Ki-67 positive

Kaplan–Meier analysis showed no significantly different survival across the four groups by Ki-67 (*P* = 0.17), Her2 (*P* = 0.62), or combined expression of Her2 and Ki-67 (*P* = 0.24; Fig. 3). Using Cox proportional hazards regression (Table 7), neither univariable nor multivariable-adjusted analysis showed a significant association of Ki-67 expression with survival; a strongly positive (+++) Her2 expression was significantly associated with poorer survival compared to − expression in multivariable analysis (hazard ratio = 2.17, 95% confidence interval = 1.01–4.68). Using the Cox model, neither univariable nor multivariable-adjusted analysis showed a significant association of combined Her2 and Ki-67 expression with survival (Table 7).

**Table 7:** Association of Her2 and Ki-67 expressions with survival using univariable and multivariable-adjusted Cox proportional hazards regression

Variable	Univariable analysis^a^		Multivariable analysis^b^	
	HR (95% CI)	*P*	HR (95% CI)	*P*
Ki-67 expression				
As continuous	1.00 (0.99–1.01)	0.929	1.00 (0.99–1.01)	0.645
−	1 (reference)		1 (reference)	
+	1.42 (0.67–3.03)	0.360	1.15 (0.47–2.84)	0.758
++	0.84 (0.41–1.74)	0.638	0.73 (0.31–1.75)	0.485
+++	1.51 (0.66–3.46)	0.326	1.46 (0.56–3.79)	0.436
Negative	1 (reference)		1 (reference)	
Positive	0.75 (0.49–1.14)	0.178	0.76 (0.48–1.19)	0.230
Her2 expression				
−	1 (reference)		1 (reference)	
+	1.25 (0.73–2.14)	0.425	1.04 (0.57–1.89)	0.897
++	1.14 (0.64–2.03)	0.651	1.27 (0.67–2.41)	0.467
+++	1.28 (0.66–2.48)	0.467	*2.17* (1.01–4.68)	*0.047*
Negative	1 (reference)		1 (reference)	
Positive	1.12 (0.71–1.76)	0.620	1.52 (0.90–2.55)	0.118
Ki-67 and Her2 expression				
Her2 negative and Ki-67 negative	1 (reference)		1 (reference)	
Her2 negative and Ki-67 positive	0.61 (0.37–1.01)	0.054	0.70 (0.41–1.20)	0.193
Her2 positive and Ki-67 negative	0.73 (0.34–1.54)	0.404	1.30 (0.56–3.01)	0.538
Her2 positive and Ki-67 positive	0.89 (0.49–1.61)	0.689	1.17 (0.60–2.29)	0.646

The positivity degree of Her2 expression follows the National Comprehensive Cancer Network (NCCN) Guidelines (https://www.nccn.org): − means “no reactivity or membranous reactivity in < 10% of cancer cells”; + means “faint or barely perceptible membranous reactivity in ≥ 10% of cancer cells; cells are reactive only in part of their membrane”; ++ means “weak to moderate complete, basolateral or lateral membranous reactivity in ≥ 10% of cancer cells”; +++ means “strong complete, basolateral, or lateral membranous reactivity in ≥ 10% of cancer cells”. – or + expression indicates a negative result, and +++ expression suggests a positive finding. In case of ++ expression by immunohistochemistry, in situ hybridization was further performed, and cases with an average Her2 copy number ≥ 6.0 signals/cell are considered positive. For Ki-67, − means very low proliferation activity with proportion of Ki-67-positive cells < 25%; + means low proliferation activity with proportion of Ki-67-positive cells 25–50%; ++ means moderate proliferation activity with proportion of Ki-67-positive cells 50–75%; +++ means high proliferation activity with proportion of Ki-67-positive cells > 75%. A section was regarded negative in Ki-67 expression with < 50% positive cells, and positive with ≥ 50% positive cells. Significant associations are shown in italics

*Her2* human epidermal growth factor receptor 2, *HR* hazard ratio, *CI* confidence interval

^a^Performed using univariable Cox proportional hazards regression

^b^Performed using multivariable Cox proportional hazards regression adjusting for sex, age, tumor location, length, differentiation grade, pT stage, and pN stage

## Discussion

Given that Her2 and Ki-67 might both exert functions in discrepant phases during cell proliferation, the combined expression of both markers were examined in our study. We found that in non-metastatic GaC, stronger expression of the receptor tyrosine kinase Her2 alone was significantly associated with better tumor differentiation, neurovascular invasion, less advanced pT stage, and more advanced pN stage; while the expression of the nucleus-associated antigen Ki-67 alone was not significantly associated with any investigated clinicopathologic factors. Patients with both negative Her2 and negative Ki-67 expression had poorer tumor differentiation, and more advanced pT and pTNM stages; the association with pT and pTNM stages were further confirmed by multivariable analyses, especially in node-negative disease. Her2 or Ki-67 alone was not significantly associated with pTNM stage. A strongly positive (+++) Her2 expression was associated with poor survival; while Ki-67 or combined expression was not significantly associated with prognosis.

The human *Her2* gene located in chromosome 17q12-21.23 can code a transmembrane receptor tyrosine kinase, and the interaction of the receptor with their ligands can modulate cell survival, growth, differentiation, and proliferation through altering cell signaling [25]. We found that it was majorly expressed in GaC cell membrane. Studies have indicated a role of Her2 in the development of various types of human cancers. The proportion of positive expression is about 10–20% in breast carcinoma [26] and is approximately 20% in GaC, where a high expression of Her2 is associated with poor prognosis [27]. A phase III randomized trial [9] showed that trastuzumab (an anti-Her2 agent) in combination with conventional chemotherapy is superior to conventional chemotherapy alone in the treatment of Her2/neu-positive advanced GaC.

In our study, stronger Her2 expression was associated with better tumor differentiation but with neurovascular invasion. Interestingly, higher Her2 expression was associated with earlier pT stage, but with more advanced pN stage. In a pooled analysis, while consistently, Her2 expression was found to be positively associated with lymph node metastasis, it was not significantly associated with cancer invasion depth or TNM stage [28]. A study [29] has shown that the expression of Her2 is not the result of the development and progression of malignant tumor, but rather the initiating carcinogenic factor of the disease. This might partly explain the observed association with the primary tumor stage. The positive association between Her2 expression and involved lymph node number suggests that Her2 may play an important role in the nodal metastasis of GaC. The role of Her2 in the progression of GaC warrants further exploration. It is well known that in breast cancer Her2 was found to be a negative prognostic factor [30], but for GaC there still seems to be no consensus, despite the fact that the first studies demonstrated an association between positive Her2 status and poor prognosis. The majority of previous publications showed that Her2-postive status provided additional prognostic information and was associated with relevant clinicopathologic characteristics, such as serosal invasion and lymph node metastasis [31–33]. We found that Her2 expression was significantly associated with tumor pT and pN stages, and differentiation with some seemingly contradictory patterns, and that only a strongly positive (+++) expression of Her2 was marginally significantly associated with poorer survival.

Ki-67 is receiving increasing attention as an important tumor proliferation marker since firstly revealed as a non-histone protein in 1991 by Gerdes et al. [34], and is highly associated with tumor development, progression, invasion, metastasis, and prognosis [35]. It is only expressed in the nucleus of proliferating cells during the proliferation and synthesis phases of the cell cycle, and could be detected from the G1 through the M phase, but not in the resting G0 phase. It has been used as a proliferation marker in cancers [36]. Some studies screening potential tumor markers and investigating their correlation with clinicopathologic factors have shown that Ki-67 is associated with tumor invasion and metastasis in various cancer types [10–12]. A study [37] suggests that Ki-67 is an independent prognostic factor for breast cancer patients with positive sentinel lymph nodes who have received adjuvant chemotherapy.

This study revealed that the expression of Ki-67 alone was not significantly associated with any investigated clinicopathologic factors. Studies on the association between Ki-67 expression and clinicopathologic factors revealed controversial results [38–40]. Notably, a recent meta-analysis showed that high Ki-67 expression was not significantly associated with lymph node metastasis, tumor stage, or differentiation, but that it could serve as a predictive biomarker for poor prognosis in GaC patients [41]. However, our study further showed that Ki-67 expression was also not significantly associated with postsurgical survival.

Her2 or Ki-67 alone has ambiguous clinicopathologic significance. We here for the first time revealed the significance of the combined examination of both markers in GaC. Based on the significance of individual markers [25, 29, 35, 36], it is hypothesized that in non-metastatic GaC, cancers with negative expression of both markers may be relatively weak in both local growth and nodal metastasis capacities, cancers with positive Ki-67 expression but negative Her2 expression may have a stronger local growth potential, tumors with positive Her2 expression but negative Ki-67 expression may be more prone to invasion and nodal metastasis, and tumors with positive expression of both markers may be strong in both capacities. GaC negative in both marker expression can be more heterogeneous and can also include quite a proportion of more advanced cancers where the biological role of local growth and nodal involvement has been relatively weakened during the specific phase of malignant progression. The biological relevance needs to be further clarified in translational or basic studies. In our study, patients with both negative Her2 and Ki-67 expression had poorer tumor differentiation, and more advanced pT and pTNM stages, especially in node-negative disease. The combined expression of both markers was not significantly associated with pN stage. The discrepancy in the associations of the marker expression with tumor local invasion and with lymph node metastasis and the seemingly contradictory findings between the associations of Her2 and Ki-67 expression with cliniopathologic parameters and with survival should be better clarified in future studies. Notably, the assessment of Ki-67 in archived tissue samples is unsuitable as a prognostic biomarker for GaC. Ki-67 expression may be complicated by tumor heterogeneity, and future studies should pay special attention to standardized evaluation and appropriate and representative tissue sampling [23]. In this study the tissue samples were examined soon after resection.

This study is first limited by its observational nature. Possibly due to difference in cancer entity, proportion of positive Ki-67 expression was as high as 95% when we followed the recommendations by the International Ki-67 in Breast Cancer working group [42], which we considered not suitable for classifying Ki-67 expression in GaC. Different cutoff values for Ki-67 in GaC were adopted in previous studies [41], and there lacks a standard or a recommendation. While in this report we used the cutoff value following recent publications [20–24], the optimal one needs to be further determined. In this study, multivariable regression models were fitted when patient age and tumor length were assessed as continuous covariates to avoid arbitrary selection of cut-off points for continuous variables.

The novelty of this work lies in the fact that the combined expression of Her2 and Ki-67 was examined, in addition to either individual marker. While there have been investigations on the clinical significance of Her2 expression in patients with gastric cancer, they mostly investigated Her2 alone, and a large proportion of the studies were on advanced unresectable gastric cancer; furthermore, the associations of Her2 expression with clinicopathologic factors (especially tumor pTNM stage, pT stage, pN stage, and differentiation) and prognosis remain largely controversial, and the quality of statistics in previous studies varied [13, 28]. In this study, we investigated the significance of Her2 expression in resected gastric adenocarcinoma without distant metastasis in a cohort study design, both alone and in comparison to and in combination with Ki-67 expression, using both univariable and multivariable analyses. We performed careful analyses of the association of Her2 expression with various clinicopathologic factors especially cancer stages both overall and with careful subgroup analyses stratified by lymph node metastasis status. The findings also aid to personalized medicine and provide important hints for further investigation. Cancer genesis and progression are both dependent on cell proliferation, and Her2 and Ki-67 are expressed in proliferating cells during various phases. Both play vital regulatory roles in cell proliferation as major proliferation indexes [43]. Other strengths of our study included the strict inclusion and exclusion criteria, the careful cohort study design, and the careful statistical and subgroup analyses.

## Conclusions

In non-metastatic GaC, Her2 expression and combined expression of Her2 and Ki-67 were associated with several clinicopathologic factors including tumor differentiation and stage, and only a +++ Her2 expression was associated with poorer prognosis in multivariable analysis with marginal significance in this study; while Ki-67 alone had both limited clinicopathologic and prognostic values.

## Data Availability

Restrictions apply to the availability of the data that support the findings of this study, which were used under license for the current study, and so are not publicly available. However, the data used/generated by our study is available from the corresponding author upon reasonable request, and formal study proposal and variable sheet and approval by the authors’ institution are needed.

## Abbreviations

GaC: Gastric adenocarcinoma
TNM stage: Tumor-node-metastasis stage
pTNM stage: Pathological tumor-node-metastasis stage
pT stage: Pathological tumor stage
pN: Pathological node stage
Her2: Human epidermal growth factor receptor 2
